# 抗血管生成药物联合免疫检查点抑制剂治疗晚期非小细胞肺癌的研究进展

**DOI:** 10.3779/j.issn.1009-3419.2021.101.05

**Published:** 2021-03-20

**Authors:** 婧怡 王, 文颖 彭, 美林 江, 麟 邬

**Affiliations:** 410013 长沙，中南大学湘雅医学院附属肿瘤医院/湖南省肿瘤医院胸部内二科 The Second Department of Thoracic Oncology, the Affiliated Cancer Hospital of Xiangya School of Medicine, Central South University/Hunan Cancer Hospital, Changsha 410013, China

**Keywords:** 肺肿瘤, 抗血管生成药物, 免疫检查点抑制剂, 肿瘤免疫微环境, Lung neoplasms, Anti-angiogenic agents, Immune checkpoint inhibitor, Tumor immune microenvironment

## Abstract

肺癌是中国乃至全世界发病率和死亡率最高的癌种，其中非小细胞肺癌（non-small cell lung cancer, NSCLC）约占85%。肿瘤的生长和转移依赖于肿瘤新生血管的形成，抗血管生成治疗的地位日益显著，但仅接受抗血管生成单药治疗无法使患者预后明显改善。近年来，免疫检查点抑制剂（immune checkpoint inhibitor, ICI）的应用显著地改善了部分肺癌患者的预后，但接受ICI单药治疗人群的缓解率较低，而抗血管生成药物和免疫检查点抑制剂均能调节肿瘤微环境、有潜在协同作用机制，联合应用于抗肿瘤治疗有较好前景。本文将就抗血管生成药物联合免疫检查点抑制剂在晚期NSCLC中的研究及应用进行综述。

## Abstract (en)

Lung cancer has the highest incidence rate and mortality in China, even in the world, and non-small cell lung cancer (NSCLC) accounts for about 85%. The growth and metastasis of tumor depend on the generation of blood vessels, and anti-angiogenic therapy is playing an increasingly important role, however, no significant improvement was observed in the underwent anti-angiogenic agents used for patients alone. In recent years, the application of immune checkpoint inhibitor (ICI) has significantly improved the prognosis of some lung cancer patients, however, the objective response rate of patients receiving ICI alone is low. While anti-angiogenic agents and ICI both regulate the tumor immune microenvironment and have a potential synergistic mechanism, showing a bright prospect in the combined application of anti-tumor therapy. In this review, we focused on the research and application of anti-angiogenic agents in combination with ICI in advanced non-small cell lung cancer.

GLOBOCAN2018癌症报告^[1]^显示，肺癌是中国以及全世界发病率最高和死亡率最高的癌种，其中非小细胞肺癌（non-small cell lung cancer, NSCLC）约占85%，是肺癌的主要组织学类型，大多数患者就诊时已处于中晚期。近年来肺癌精准治疗进展迅速，靶向治疗、抗血管生成治疗及免疫治疗等精准治疗方式层出不穷。肿瘤的生长和转移依赖于肿瘤新生血管的形成，抗血管生成治疗逐渐引起人们关注，但接受抗血管生成单药治疗的患者并未得到明显预后改善。随着程序性死亡受体1（programmed cell death 1, PD-1）抑制剂、程序性死亡受体配体1（programmed cell death ligand 1, PD-L1）抑制剂、细胞毒性T淋巴细胞相关抗原4（cytotoxic T lymphocyte-associated antigen 4, CTLA-4）抑制剂等相关药物获批，免疫检查点抑制剂（immune checkpoint inhibitor, ICI）已成为肺癌领域的研究热点，很大程度地改善了部分肺癌患者的预后，但接受ICI单药治疗人群的缓解率较低，如何扩大免疫治疗响应人群是目前亟待解决的问题^[2]^。免疫治疗的响应和肿瘤微环境（tumor microenvironment, TME）的免疫浸润状态有关，而抗血管生成药物治疗不仅参与微环境中异常血管的重构，调节肿瘤免疫细胞浸润，还能逆转TME的免疫抑制状态^[3]^。因此，本文将阐述抗血管生成药物联合免疫检查点抑制剂在晚期NSCLC中的应用，分析联合治疗的作用机制，汇总相关临床研究的最新进展，分析该联合模式在晚期NSCLC患者中应用的可能性及前景。

## 1 抗血管生成药物联合ICI的抗肿瘤机制

### 1.1 抗血管生成药物的抗肿瘤机制

1971年Folkman首次提出了“肿瘤的生长和转移依赖于血管生成”的假说^[4]^。由于肿瘤细胞的快速分裂和生长会消耗大量氧气和营养，而肿瘤微环境低氧可诱导肿瘤和基质细胞分泌多种促血管生成因子，导致多个血管生成途径被激活^[5]^。其中，血管内皮生长因子A（vascular endothelial growth factor A, VEGF-A）为主要的促血管生成因子，其主要的功能性受体为血管内皮生长因子受体2（vascular endothelial growth factor receptor 2, VEGFR-2）^[6, 7]^。而抗血管生成药物可以通过阻断新生血管生成，逆转TME的免疫抑制状态，从而起到抗肿瘤的作用。

### 1.2 ICI的抗肿瘤机制

免疫检查点是人体免疫系统中起抑制作用的调节分子，可以防止T细胞过度激活导致机体炎症损伤，而肿瘤细胞利用高表达的免疫检查点分子发生免疫逃逸^[8]^。目前研究相对深入的免疫检查点有PD-1^[9]^和CTLA-4^[10]^等。

PD-1/PD-L1是T细胞活化的负调控信号通路，PD-1/PD-L1抑制剂通过阻断该通路，使受抑制的T细胞重新活化，增强其对肿瘤抗原的识别和对肿瘤细胞的杀伤^[11]^。CTLA-4是另一种负性调节T细胞活化的共刺激分子，CTLA-4抑制剂能有效阻断CTLA-4与B7分子的结合，恢复共刺激信号通路CD28-B7的活性，减弱对T细胞活化的抑制作用，增加肿瘤特异性T细胞的浸润^[12, 13]^。

### 1.3 抗血管生成药物联合ICI的抗肿瘤机制

免疫治疗的疗效在不同患者中的差异很大，研究^[14]^认为可能与TME中免疫细胞浸润的异质性有关。对于大多数实体瘤，肿瘤异常血管生成以多种方式影响TME中的免疫细胞浸润和功能，导致免疫抑制型微环境。新生的肿瘤血管形态结构异常，相邻内皮细胞间连接松散，导致血管易渗漏^[15]^，并且高水平的VEGF可以阻断血管内皮细胞黏附分子的表达^[16]^，从而减少TME中免疫细胞的黏附和浸润; 而异常肿瘤血管灌注能力受损，进一步导致TME缺氧加重，使肿瘤浸润淋巴细胞的功能受到损害^[17-22]^。而抗血管生成药物可以通过多种途径改善免疫细胞浸润，逆转TME的免疫抑制状态，进而协同增强ICI的疗效^[3, 23]^。合理的抗血管生成药物剂量和给药时间^[24]^可以通过抑制肿瘤血管的异常生成、改善血管周细胞覆盖率及促进血管成熟，来诱导肿瘤血管正常化，正常的血管网络可以直接促进免疫细胞的黏附、浸润。血管灌注能力的恢复可以缓解TME缺氧，减少VEGF的分泌，从而减少免疫抑制性细胞如骨髓源性抑制细胞和调节性T细胞的募集^[17]^，减少腺苷和乳酸等免疫抑制性代谢物在TME中的积累和改善酸中毒^[18]^，并能诱导肿瘤相关巨噬细胞极化为免疫支持的M1样表型^[19]^，还可以降低免疫抑制细胞表面PD-L1、CTLA-4、T细胞免疫球蛋白黏蛋白-3（T cell immunoglobulin domain and mucin domain-3, TIM-3）等免疫检查点分子的表达^[20, 21]^和减少VEGF、转化生长因子-β（transforminggrowthfactor-β, TGF-β）、白细胞介素10（Interleukin 10, IL-10）等免疫抑制因子的分泌^[22]^，进而恢复免疫细胞的活化及功能。抗血管生成药物还可以通过阻断VEGF和巨噬细胞、T细胞表面的VEGFR-2结合，促进免疫细胞成熟和改善浸润^[25-27]^。

同时，ICI不仅可以调节免疫微环境，还可以抑制异常肿瘤血管生成。ICI通过激活免疫效应细胞，活化的CD4^+^ T细胞、CD8^+^ T细胞、Th1细胞等分泌INF-γ、CXCL9、CXCL10和TNF等抗肿瘤细胞因子，发挥抗肿瘤异常血管生成、促进血管正常化的作用^[28, 29]^。

综上，抗血管生成药物可以改善TME中的免疫细胞浸润状态，从而协同ICI的疗效; 而ICI既可以激活免疫细胞，又可以进一步促进血管正常化和TME重塑，最终促成长期的肿瘤控制。理论上，抗血管生成治疗和ICI治疗联合，使血管正常化和免疫重构之间形成正反馈回路，为临床上二者的联合使用提供了支持。

## 2 抗血管生成药物联合ICI的临床研究进展

近年来，一些旨在评估抗血管生成药物联合ICI治疗晚期NSCLC的有效性和安全性的临床研究已公布结果，目前数据初步表明，这种联合治疗模式具有良好的应用前景（表 1）。

**1:** 抗血管生成联合ICI治疗晚期NSCLC临床研究数据总结 Summary of clinical trials of anti-angiogenic agents combined with ICI in NSCLC

Study	IMpower150 NCT02366143	Be study	JVDF NCT02443324	NCT02501096	LEAP-006 NCT03829319	NCT03628521	NCT03083041
Design	Phase 3 Randomized	Phase 2 Single group	Phase 1 Non-randomized	Phase 1b/2 Single group	Phase 3 Randomized Double-blind	Phase 1 Non-randomized	Phase 2 Single group
Patient	Stage Ⅳ non-squamous NSCLC	Stage Ⅳ NSCLC with high PD-L1 expression	Stage Ⅳ NSCLC	Stage Ⅳ NSCLC	Stage Ⅳ non-squamous NSCLC	Stage Ⅳ NSCLC	Stage Ⅲb/Ⅳ NSCLC
Arm	Atezo+PacC Bev+Atezo+PacC Bev+PacC	Bev+Atezo	Ram+Pembro	Lenva+Pembro	Lenva+Pembro+Pem+Pla Placebo+Pembro+Pem+Pla	Anlo+Sinti	Apa+Camre
Sample size	*n*=1, 202 ACP: 402 ABCP: 400 BCP: 400	*n*=39	*n*=26	*n*=21	*n*=13 (part 1)	*n*=22	*n*=91 (Non-squamous NSCLC) *n*=25 (Squamous NSCLC)
Primary endpoint	PFS in Teff-high WT PFS in ITT-WT OS in ITT-WT	ORR	Safety	ORR at 24 weeks	Safety	Safety ORR	Safety ORR
Result	ABCP *vs* BCP mPFS: 11.3 mon *vs* 6.8 mon *P* < 0.001 (Teff-high WT) mPFS: 8.3 mon *vs* 6.8 mon *P*=0.01 (ITT-WT) mOS: 19.5 mon *vs* 14.7 mon *P* < 0.001 (ITT-WT)	ORR: 64.1% One year PFS rate: 54.9%	ORR: 42.3% DCR: 84.6% mPFS: 9.3 mon mOS: NR	ORRWK24: 33.3% DCR: 80.9% mPFS: 7.4 mon	ORR: 69.2% DCR: 92.3%	ORR: 77.3% DCR: 100%	ORR: 30.8%; DCR: 82.4% mPFS: 5.9 mon; mOS: NR (Non-squamous NSCLC) ORR: 32%; DCR: 84% mPFS: 6 mon; mOS: 12.8 mon (Squamous NSCLC)
TRAEs	ABCP *vs* BCP Grade 3/4: 55.7% *vs* 47.7% Grade 5: 2.8% *vs* 2.3%	NA	≥Grade 3: 42.3%	Grade 3: 48% Grade 4: 5%	≥Grade 3: 77%	≥Grade 3: 31.8%	≥Grade 3: 67.5% (Non-squamous NSCLC) NA (Squamous NSCLC)
Bev: bevacizumab; Ram: ramucirumab; Anlo: anlotinib; Apa: apatinib; Lenva: lenvatinib; Pembro: pembrolizumab; Atezo: atezolizumab; Sinti: sintilimab; Camre: camrelizumab; Pem: pemetrexed; PacC: paclitaxel plus carboplatin; Pla: Platinum; NSCLC: non-small cell lung cancer; PD-L1: programmed cell death ligand 1; Teff-high: effector T cell high; ITT: intention to treat; WT-IIT: wild type intention to treat; Mo: month; PFS: progression-free survival; mPFS: median progression-free survival; OS: overall survival; mOS: median overall survival; ORR: objective response rate; ORRWK24: objective response rate at 24 weeks; DCR: disease control rate; TRAEs: treatment-related adverse events; NR: not reach; NA: not applicable; ICI: immune checkpoint inhibitor.							

### 2.1 抗VEGFR单克隆抗体联合ICI

贝伐珠单抗是一种重组人源化抗VEGFR单克隆抗体，可阻断VEGF-A与其受体VEGFR-1及VEGFR-2的结合。

IMpower150研究（NCT02366143）^[30, 31]^是首个抗血管生成药物联合ICI一线治疗晚期NSCLC有无疾病进展生存时间（progression-free survival, PFS）和总生存期（overall survival, OS）统计学获益的Ⅲ期临床研究。该研究共纳入1, 202例初治的Ⅳ期或复发转移的非鳞状NSCLC患者，按1:1:1随机分配接受阿特珠单抗+卡铂+紫杉醇治疗（ACP组402例），或阿特珠单抗+贝伐珠单抗+卡铂+紫杉醇治疗（ABCP组400例），或贝伐珠单抗+卡铂+紫杉醇治疗（BCP组400例）。研究结果显示，在EGFR/ALK野生型患者中，相较于BCP组，ABCP组PFS和OS显著获益（中位PFS：8.3个月*vs* 6.8个月，HR=0.62，*P* < 0.001;中位OS：19.5个月*vs* 14.7个月，HR=0.80，*P*=0.01），达到了主要研究终点。在全部患者中，ABCP组相比于BCP组，PFS和OS也显著延长（中位PFS：8.4个月*vs* 6.8个月，HR=0.59，*P* < 0.001;中位OS：19.8个月*vs* 14.9个月，HR=0.76，*P*=0.01），客观缓解率（objective response rate, ORR）和缓解持续时间（duration of response, DoR）也均优于BCP组（ORR：56% *vs* 40%;DoR：11.5个月*vs* 6.0个月），达到了次要研究终点。在亚组分析中，对各分层亚组如：不同效应T细胞基因表达谱水平、不同PD-L1表达水平、*EGFR*突变阳性、肝转移的晚期NSCLC患者，ABCP组相对于BCP组也均有不同程度获益。在安全性方面，两组整体治疗相关不良反应发生率无明显差异，且未出现新的不良事件，与既往报道的安全性相似。基于IMpower150的研究结果，ABCP四药联合方案已成为美国国家综合癌症网络指南中非鳞NSCLC的一线治疗推荐，有望成为未来的主流治疗方案，证明了抗血管生成药物联合ICI在晚期NSCLC中具有良好应用前景。而目前正在开展的IMpower151研究，旨在探索ABCP方案在中国人群晚期NSCLC中的疗效及安全性，期待未来有更多的抗血管生成药物联合ICI方案在中国人群获益的新证据。

此外，各种抗血管生成与免疫联合用药方案不断推陈出新，并尝试开启去化疗模式的探索。一项单臂Ⅱ期研究^[32]^进行了在PD-L1高表达非鳞NSCLC中仅运用贝伐珠单抗联合阿特珠单抗、去化疗模式的尝试。纳入分析的39例患者数据显示，该研究达到了主要研究终点，ORR为64.1%，次要研究终点如PFS、DoR、OS及安全性等数据有待进一步披露。而这种去化疗模式是否能够不劣于IMPower150研究的四药联合模式，则有待Ⅲ期随机对照研究的证实。

### 2.2 抗VEGFR-2单克隆抗体联合ICI

雷莫芦单抗是一种抗VEGFR-2单克隆抗体，可以靶向阻断VEGF与VEGFR2的结合，从而起到抗血管生成的作用。JVDF研究（NCT02443324）的NSCLC队列^[33]^共入组了26例晚期NSCLC患者，一线给予雷莫芦单抗联合帕博利珠单抗治疗。至数据截止时，总体ORR为42.3%，疾病控制率（disease control rate, DCR）为84.6%，中位PFS为9.3个月，中位OS在随访24.8个月后暂未达到，整体安全性良好，分层分析显示PD-L1高表达人群较PD-L1低表达人群疗效更佳。虽尚无雷莫芦单抗联合帕博利珠单抗与单用帕博利珠单抗的头对头试验，但JVDF研究仍显示出抗血管生成联合免疫治疗具有临床优势。

### 2.3 多靶点小分子酪氨酸激酶抑制剂（tyrosine kinase inhibitor, TKI）联合ICI

多靶点小分子TKI除了可以抑制VEGFR外，还可以抑制成纤维生长因子受体、血小板源性生长因子受体、c-Kit、Ret等^[34]^，其与ICI的联合方案在治疗晚期NSCLC中也取得了初步结果，代表药物如：仑伐替尼、安罗替尼、阿帕替尼等，都在联合免疫治疗领域进行了研究探索。

#### 2.3.1 仑伐替尼联合ICI

一项Ib期/Ⅱ期的临床试验（NCT02501096）^[35]^纳入了21例晚期NSCLC患者，接受仑伐替尼联合帕博利珠单抗治疗，其中14%为初治、33%接受过一线治疗、48%接受过二线治疗、5%接受过三线及以上治疗，总体ORR为33.3%，DCR为80.9%，中位PFS为7.4个月，且总体安全可控。基于以上，该方案在晚期NSCLC中开启了Ⅲ期临床试验（NCT03829319），LEAP-006^[36]^第一部分研究数据显示在帕博利珠单抗联合化疗的基础上加用仑伐替尼，对于初治的晚期NSCLC患者疗效确切，13例有效分析数据显示该联合模式的ORR达到69.2%，DCR为92.3%，耐受性良好，第二部分随机研究目前正在进行中，期待后续相关数据的公布。

#### 2.3.2 安罗替尼联合ICI

在探索我国原研多靶点抗血管生成小分子TKI联合ICI治疗晚期NSCLC方面，一项关于信迪利单抗联合安罗替尼作为晚期NSCLC一线治疗有疗效和安全性的Ⅰ期研究（NCT03628521）^[37]^，共纳入22例晚期NSCLC，初步结果显示所有患者接受联合治疗的耐受性均良好，≥3级的治疗相关不良反应事件发生率为31.8%，ORR为77.3%，DCR高达100%。基于患者基线时的PD-L1表达和肿瘤突变负荷（tumor mutation burden, TMB）进行亚组分析，结果显示联合治疗在各亚组中均具有一致的疗效获益。虽然数据截止时的PFS尚不成熟，但该方案展示出良好的抗肿瘤活性。

#### 2.3.3 阿帕替尼联合ICI

一项Ⅱ期研究（NCT04239443）^[38]^探索了阿帕替尼联合卡瑞利珠单抗作为晚期NSCLC二线及以上治疗方案的有效性及安全性。在91例可评估的非鳞状NSCLC受试者中，ORR为30.8%，DCR为82.4%，中位PFS为5.9个月，总生存OS未达到，分层分析显示在bTMB-high的患者中观察到了更好的临床疗效。2020年欧洲肿瘤内科学会年会上公布的数据^[39]^显示该方案在非中央型鳞状NSCLC受试者中也观察到了临床获益，在25例受试者中，ORR为32%，DCR为84%，中位PFS为6.0个月，中位OS为12.8个月，且PD-L1表达阳性患者获益更多。阿帕替尼联合卡瑞利珠单抗在非鳞状/鳞状NSCLC中均提升了二线治疗的有效率和疗效，该联合方案一线治疗PD-L1表达阳性NSCLC的Ⅲ期临床研究（NCT04203485）正在开展，希望未来能为患者提供新的一线治疗方案。

### 2.4 其他

抗血管生成药物联合ICI的临床研究逐渐增多，表 2为登记在Clinical Trail网站（https://www.clinicaltrials.gov）上的临床试验汇总（截止2021年2月2日）。

**2:** 抗血管生成药物联合免疫治疗晚期NSCLC的临床试验 Clinical trials of anti-angiogenic agents combined with immunotherapy in advanced NSCLC

Clinical trial	Treatment	Patient	Design
mAbs targeting VEGF-VEGFR combined with ICI			
First line			
NCT02039674	Bev+Pembro+PacC	Stage Ⅲb/Ⅳ NSCLC	Phase 1/2, randomized
NCT01454102	Bev+Nivo	Stage Ⅲb/Ⅳ NSCLC	Phase 1, randomized
NCT02574078	Bev+Nivo	Stage Ⅳ NSCLC	Phase 1/2, randomized
NCT02366143 (Impower 150)	Bev+Atezo+PacC Atezo+PacC Bev+PacC	Stage Ⅳ chemotherapy-naive non-squamous NSCLC	Phase 3, randomized
NCT03836066	Bev+Atezo	Stage Ⅲb/Ⅳ high-intermediate TMB selected non-squamous NSCLC	Phase 2, single group
NCT03713944	Bev+Atezo+PemC	Stage Ⅳ non-squamous NSCLC	Phase 2, single group
NCT04194203 (IMpower151)	Bev+Atezo+PacC/PemC Bev+Placebo+ PacC/PemC	Stage Ⅳ Chemotherapy-naive non-squamous NSCLC	Phase 3, randomized, double-blind
Second line or beyond			
NCT03647956	Bev+Atezo+PemC	Stage Ⅳ *EGFR*-mutant NSCLC after failure of *EGFR*-TKIs	Phase 2, single group
NCT04042558	Bev+Atezo+PemC Atezo+PemC	Stage Ⅲb/Ⅳ non-squamous NSCLC with progression-enhancing mutations following targeted therapies	Phase 2, randomized
NCT04245085 (ABC-lung)	Bev+Atezo+PacC Bev+Atezo+Pem	Stage IIIb/Ⅲc/Ⅳ *EGFR*-mutant NSCLC with acquired resistance	Phase 2, randomized
NCT04426825	Bev+Atezo	Stage Ⅲb/Ⅳ *EGFR*-mutant non-squamous NSCLC after failure of *EGFR*-TKIs	Phase 2, single group
NCT04099836	Bev+Atezo	Stage Ⅳ *EGFR*-mutant NSCLC after failure of Osimertinib	Phase 2, single group
NCT03991403	Bev+Atezo+PacC Pem+Cis/Car	Stage Ⅲb/Ⅳ non-squamous NSCLC with *EGFR* mutation or ALK translocation after failure of TKIs	Phase 3, randomized
NCT04213170	Bev+Sinti	Stage Ⅳ driving gene-negative NSCLC with asymptomatic brain metastases	Phase 2, single group
NCT03971474	Ram+Pembro Ram/Pem/Doc/Gem	Stage Ⅳ NSCLC previously treated with ICI	Phase 2, randomized
NCT04120454	Ram+Pembro	Stage Ⅳ *EGFR*-mutant NSCLC after failure of *EGFR*-TKIs	Phase 2, single group
NCT04340882	Ram+Pembro+Doc	Stage Ⅳ NSCLC progressed on platinum-doublet and PD-1/PD-L1 blockade	Phase 2, single group
NCT03689855	Ram+Atezo	Stage Ⅳ NSCLC previously treated with ICI	Phase 2, single group
NCT02572687	Ram+Durva	Stage Ⅲb/Ⅳ NSCLC previously treated with systemic therapy	Phase 1, non-randomized
Unlimited			
NCT02681549	Bev+Pembro	Stage Ⅳ NSCLC with untreated brain metastases	Phase 2, single group
NCT03786692	Bev+Atezo+PemC Bev+PemC	Stage Ⅳ non-squamous NSCLC with sensitizing *EGFR* mutation and never smoked	Phase 2, randomized
NCT02443324	Ram+Pembro	Stage Ⅲb/Ⅳ NSCLC	Phase 1, non-randomized
Small molecule TKI combined with ICI			
First line			
NCT04164745	Anlo+Pembro	Stage Ⅳ PD-L1 positive treatment-naive NSCLC	Phase 2, single group
NCT03628521	Anlo+Sinti Anlo+Erlo Anlo+ PemC/GemC	Stage Ⅳ NSCLC	Phase 1, non-randomized
NCT03829319	Lenva+Pembro+Pem+Pla Placebo+Pembro+Pem+Pla	Stage Ⅳ non-squamous NSCLC	Phase 3, randomized, double-blind
NCT03829332	Lenva+Pembro Placebo+Pembro	Stage Ⅳ PD-L1 positive treatment-naive NSCLC	Phase 3, randomized, double-blind
NCT03516981	Lenva+Pembro MK-1308+Pembro MK-4280+Pembro	Stage Ⅳ NSCLC	Phase 2, randomized
Second line or beyond			
NCT04165330	Anlo+Nivo	Stage Ⅲb/Ⅳ NSCLC after at least one prior line of standard therapy	Phase 1/2a, single group
NCT03765775	Anlo+Sinti	Stage Ⅳ NSCLC received first-generation *EGFR*-TKIs resistance along with T790M negative	Phase 2, single group
NCT04316351	Anlo+Tripa+Pem	Stage Ⅲb/Ⅳ T790M positive NSCLC after Osimertinib resistance	Phase 2, single group
NCT04239443	Apa+Camre Camre PacC/PemC	Stage Ⅳ NSCLC	Phase 2, single group
NCT03377023	Ninte+Nivo+Ipi	Stage Ⅳ NSCLC	Phase 1/2, non-randomized
NCT04046614	Ninte+Nivo	Stage Ⅳ NSCLC of adenocarcinoma histology after one or two previous lines of systemic therapy	Phase 1/2, single group
NCT03976375	Lenva+Pembro Lenva Doc	Stage Ⅳ NSCLC after platinum doublet chemotherapy and immunotherapy	Phase 3, randomized
NCT02501096	Lenva+Pembro	Stage Ⅳ NSCLC after treatment with approved therapies	Phase 1b/2, single group
Recombinant human endostatin combined with ICI			
NCT04063449	Endo+Sinti+PemC	Stage Ⅳ non-squamous NSCLC With negative driving gene	Not applicable
NCT04303130	Endo+Camre	Stage Ⅳ squamous NSCLC	Phase 2, single group
mAbs: monoclonal antibodies; VEGF: vascular endothelial growth factor; VEGFR: vascular endothelial growth factor receptor; Bev: bevacizumab; Ram: ramucirumab; Anlo: anlotinib; Apa: apatinib; Ninte: nintedanib; Lenva: lenvatinib; Endo: endostar; Pembro: pembrolizumab; Nivo: nivolumab; Durva: durvalumab; Sinti: sintilimab; Tripa: tripalimab; Camre: camrelizumab; Ipi: ipilimumab; Pac: paclitaxel; Pem: pemetrexed; Doc: docetaxel; Gem: gemcitabine; PacC: paclitaxel plus carboplatin; PemC: pemetrexed plus carboplatin; GemC: gemcitabine plus carboplatin; Cis/Car: Cisplatin or Carboplatin; Pla: Platinum; EGFR: epidermal growth factor receptor; ALK: anaplastic lymphoma kinase; TKI: tyrosine kinase inhibitor; PD-1: programmed cell death 1; TMB: tumor mutation burden; ICI: immune checkpoint inhibitor.			

## 3 现状与挑战

目前在开展的抗血管生成药物联合ICI治疗晚期NSCLC的临床研究中，一线及后线治疗均有涉及。已公布的部分数据显示，无论在一线还是后线，联合方案均具有良好的抗肿瘤活性及临床应用前景，且总体安全性良好（表 1）。其他目前尚未公布结果的研究（表 2）大部分为Ⅰ期/Ⅱ期探索性研究，其结果及进一步Ⅲ期研究的开展值得期待，免疫治疗与抗血管生成治疗的有机结合有望为晚期NSCLC患者提供新的治疗选择，尤其是新型的ICI类药物的加入、抗血管生成与ICI双抗类新药物的研发为该领域的治疗前景提供了新的希望。在研究方案设计中，除了抗血管生成药物联合ICI，大部分研究还涉及与化疗联合，而联合化疗势必会增加毒性，在晚期NSCLC中仅运用抗血管生成药物联合ICI、“去化疗”的治疗模式是否能成为未来探索的目标和方向，还需要更多的研究数据支持; 此外，关于抗血管生成药物与ICI联合的模式到底是大分子单抗好还是小分子的TKI好，目前数据还不足以做出判断，亟待头对头的临床试验验证; 同时，我们应当注意到目前抗血管生成药物联合ICI的治疗模式仅仅是简单的药物叠加，是否还有更好的联合模式和剂量选择值得我们期待; 我们也看到有研究关注到了靶向治疗失败的驱动基因阳性患者的后线治疗，对于驱动基因阳性的NSCLC患者，抗血管生成药物联合ICI是否能成为靶向耐药后的有效治疗方案也值得探索; 而进一步在晚期NSCLC队列中探索预测联合治疗疗效的生物标志物及其潜在预测机制，也是抗血管生成药物联合ICI临床应用方向亟待解决的问题。

## 4 小结与展望

一系列临床前研究表明，抗血管生成药物和ICI具有协同抗肿瘤作用，一方面，抗血管生成药物可以通过免疫重编程逆转TME的免疫抑制状态，增强ICI的疗效，另一方面，ICI可恢复免疫支持微环境，促进血管正常化，增强抗血管生成药物的疗效。根据目前临床研究数据来看，抗血管生成药物联合ICI有望使晚期NSCLC患者临床获益、预后改善，且安全性可耐受。而探索更优药物联合方案、优化药物使用剂量和给药时间及顺序，寻找预测疗效的相关生物标志物以筛选相应优势人群等，也是未来研究的方向。综上，抗血管生成药物联合ICI在治疗晚期NSCLC患者、改善患者预后方面具有良好的应用前景。
